# River co-learning arenas: principles and practices for transdisciplinary knowledge co-creation and multi-scalar (inter)action

**DOI:** 10.1080/13549839.2024.2428215

**Published:** 2024-11-19

**Authors:** Daniele Tubino de Souza, Lena Hommes, Arjen Wals, Jaime Hoogesteger, Rutgerd Boelens, Bibiana Duarte-Abadía, Juan Pablo Hidalgo-Bastidas, Edward Huijbens, Leila M. Harris, Diana Suhardiman, Lieke Melsen, Tom Buijse, Fabio de Castro, Leontien Cremers, Barbara Hogenboom, Mariela Garcia Vargas, Denisse Roca-Servat, Gert Jan Veldwisch, K. J. Joy

**Affiliations:** a Water Resources Management Group, Department of Environmental Sciences, Wageningen University, Wageningen, Netherlands; b Department of Geography & Institute of the Environment, University of Girona, Girona, Spain; c Education and Learning Sciences, Department of Social Sciences, Wageningen University, Wageningen, Netherlands; d Norwegian Life Sciences University, Ås, Norway; e CEDLA - Center for Latin American Research and Documentation, University of Amsterdam, Amsterdam, Netherlands; f Cultural Geography Group, Department of Environmental Sciences, Wageningen University, Wageningen, Netherlands; g Institute for Resources Environment and Sustainability, University of British Columbia, Vancouver, Canada; h Department of Anthropology and Development Studies, University of Johannesburg, Johannesburg, South Africa; i Royal Netherlands Institute of Southeast Asian and Caribbean Studies, Leiden, Netherlands; j Hydrology and Quantitative Water Management Group, Department of Environmental Sciences, Wageningen University, Wageningen, Netherlands; k Aquaculture and Fisheries Group, Department of Animal Sciences, Wageningen University, Wageningen, Netherlands; l Universidad del Valle, Instituto Cinara, Cali, Colombia; m Instituto de Estudios Regionales INER at Universidad de Antioquia, Medellín, Colombia; n SOPPECOM - Society for Promoting Participative Ecosystem Management, Pune, India

**Keywords:** River regeneration, socio-ecological justice, transgressive learning, activist research, river co-learning arenas

## Abstract

This paper develops the methodological concept of river co-learning arenas (RCAs) and explores their potential to strengthen innovative grassroots river initiatives, enliven river commons, regenerate river ecologies, and foster greater socio-ecological justice. The integrity of river systems has been threatened in profound ways over the last century. Pollution, damming, canalisation, and water grabbing are some examples of pressures threatening the entwined lifeworlds of human and non-human communities that depend on riverine systems. Finding ways to reverse the trends of environmental degradation demands complex spatial–temporal, political, and institutional articulations across different levels of governance (from local to global) and among a plurality of actors who operate from diverse spheres of knowledge and systems of practice, and who have distinct capacities to affect decision-making. In this context, grassroots river initiatives worldwide use new multi-actor and multi-level dialogue arenas to develop proposals for river regeneration and promote social-ecological justice in opposition to dominant technocratic-hydraulic development strategies. This paper conceptualises these spaces of dialogue and action as RCAs and critically reflects on ways of organising and supporting RCAs while facilitating their cross-fertilisation in transdisciplinary practice. By integrating studies, debates, and theories from diverse disciplines, we generate multi-faceted insights and present cornerstones for the engagement with and/or enaction of RCAs. This encompasses five main themes central to RCAs: (1) River knowledge encounters and truth regimes, (2) transgressive co-learning, (3) confrontation and collaboration dynamics, (4) ongoing reflexivity, (5) transcultural knowledge assemblages and translocal bridging of rooted knowledge.

## Introduction

The integrity of river systems has been threatened in various ways over the last century. Pollution, damming, canalisation, unplanned occupation of precarious and flood-prone river banks, and water grabbing represent different and interlinked threats (Arthington et al. 2018; McCully 2001; Nixon 2010). Often, this environmental degradation is closely tied to social injustices, reflecting and reproducing unequal power relations (McCulligh, Arellano-García, and Casas-Beltrán 2020). As such, the lives and livelihoods of riverine communities and ecologies have been under threat.

Importantly, it is often the already disadvantaged groups that are most severely affected by deteriorating river systems or enclosures thereof, reinforcing and also exacerbating environmental and social injustices. For example, poor urban neighbourhoods are at times the last recipients along a network to receive river waters (Schmidt and Peppard 2014; Swyngedouw 2015). Others lose river access over time: people sharing cultures to whom the river is a place for spiritual and ritual worship, outdoor-enthusiasts to whom the river is the recreational landscape, or youth who practice river-based learning with nature (Souza et al. 2021; Wantzen et al. 2016). Further, villages are displaced for hydropower development (Bakker and Hendriks 2019; Boelens, Shah, and Bruins 2019), and smallholder communities lose access to sufficient irrigation and drinking water (Hommes et al. 2019; Hoogesteger and Verzijl 2015).

In general, current river governance has been largely ineffective in protecting nature and vulnerable people (UNEP 2016). Next to damming rivers and transforming water into a resource to be exploited in deeply commodified production systems, we see the classic (often colonial, and still prevalent) conservationist response: interventions that call for “dehumanizing nature”, creating exclusionary river/nature reserves (Büscher and Fletcher 2020; Latour et al. 2018; Beattie and Morgan 2017). This recreates the “enclosure of the commons” drama that has led to considerable exclusion, inequalities, and livelihood insecurity worldwide (Duarte-Abadía, Boelens, and Buitrago 2021; Harvey 1996; de Castro, Hogenboom, and Baud 2016; Hogenboom 2019).

In response to these threats, new water justice movements (NWJMs) and river co-governance initiatives are emerging to protect rivers and revitalise waters. In various locations around the world, these movements propose alternative ways of being with and acting in socio-ecological riverine systems to reverse the trends of environmental degradation. NWJMs are coalitions that bring together diverse river actors such as riverine communities, citizen initiatives, environmental organisations, as well as supralocal networks (Boelens et al. 2023). These coalitions can exist at different levels of governance. In some countries NWJMs have advocated for granting legal personhood to rivers (e.g. Australia, New Zealand, Colombia, and India; see Cribb, Macpherson, and Borchgrevink 2024; Kauffman and Martin 2018; O’Donnell and Talbot-Jones 2018), in other places they foster re-wilding, dam removal, or mobilisation against ongoing dam building projects (Sneddon, Barraud, and Germaine 2017; Del Bene, Scheidel, and Temper 2018; Villamayor-Tomas and García-López 2018; Harrington and Cantor 2024; Vos 2024). As we explore further below, at times these efforts devise creative, place – and history-based technologies, and practices for living with rivers as shared socio-ecological commons (Illich 1979; Gregory 2012; Anderson et al. 2019; Jackson 2022; Reyes Escate, Hoogesteger, and Boelens 2022). Rather than separating “natural” rivers from human society (cf. Lavau 2013; Nygren 2021), they emphasise nature-society entanglements and interdependencies (Bakker 2012; Hikuroa et al. 2021; Flaminio 2021).

While we see this multitude of initiatives, there are limited studies of their actions, claims, links, methodologies, and impact (Hommes, Vos, and Boelens 2023). This paper therefore focusses on two ongoing collaborative research projects – Riverhood and River Commons – that aim to understand and support these NWJMs and alternative river co-governance proposals. Learning from local and inter-scalar experiences, in and across different contexts and continents, the paired projects are building new conceptual and methodological tools for transformative research and stakeholder interaction. Through an action research approach, the efforts seek to enable a needed transition from commodified, mechanistic, and disconnected river systems to “commonified” and relational hydrosocial territories.

A central idea and methodology to do so is the river co-learning arena (RCA). We have coined this term to refer to multi-actor platforms that jointly conduct research, learn from grass-rooted initiatives, co-produce knowledge, and design action-oriented strategies and interventions that contribute to river regeneration and socio-ecological justice. They can take manifold forms including debate platforms, sites of struggle and disruptive action, purposeful assemblies, and/or deliberate dialogical processes. RCAs may bring together academia, local/riverine communities, indigenous peoples, public and private sectors, government agencies, and others, to promote decolonising and disruptive methodologies and learning practices.

In this paper, we draw up a framework for organising and supporting RCAs and the opportunities they can provide for strengthening grassroots movements and actions towards regenerating riverine socio-ecological systems and fostering socio-ecological justice. Importantly, our approach and thereby this paper are of a “hybrid” nature and origin: on the one hand, our contemplations are based on insights and lessons from RCA-related experiences around the world in which the authors have been involved (some experiences will be presented as illustration); on the other hand, this paper is conceptual and exploratory, drawing together insights from scholarship in the fields of sociology, anthropology, geography, political ecology, and allied domains. The objective is to reflect on what RCAs are and can be, how they can be understood and enacted. Thereby, we aim to provide a “groundwork of considerations” for organising RCAs in the future. In that sense, RCAs are not something that we have entirely implemented and completed yet. As said, different experiences have resemblances with what we envision and coin RCAs, and we draw on these experiences; at the same time we are also still in the process of developing RCAs – as presented here – in practice. This paper is thus to be used as a starting point for researchers, movements, or other initiatives that wish to develop activist research and arenas for co-learning and reflexive multi-actor interactions that integrate diverse epistemologies and ontologies.

Yet, we do not want to provide a blueprint – an undertaking that would be undesirable *and* bound to fail considering highly diverse, contextual river struggles –, but rather a number of points for reflection to be taken into account when setting up, participating in and/or studying RCAs.

Our contribution is novel in two regards. First, the paper brings the above-mentioned fields together from an action-research approach, distilling valuable multifaceted insights for the final objective (i.e. establishing, accompanying, understanding multi-actor learning-research-action platforms that contribute to river regeneration and socio-ecological justice). Second, crucially, this contribution considers multi-actor platforms (i.e. the RCAs) in their broader context – namely as part of contested socio-ecological riverscapes and struggles of/for/over power – and inquiries about the resulting conceptual, operational, and political implications. This helps to prevent the usual “participation trap” in which participation is presented and organised as depoliticised and harmonious get-together, failing to thematise and address unequal power relations, underlying politics, and broader structural issues (Turnhout et al. 2020). This perspective also provides a distinction from the increasingly popular Living Labs, which tend to emphasise collaboration and co-creation, while de-emphasising the transformative potential of conflict, disruption, and resistance (Baxter 2022).

In terms of methodology, we draw on existing scholarship, debates, and theories, and the ideas, discussions, and experiences of the diverse group of authors of this paper. We are a mixed and balanced group in terms of gender, origin and regional study focus, including scholars from a variety of disciplines, both in the social and environmental sciences, even though with slight biases: 9 out of the 19 authors being from European countries; and 10 with a regional focus on Latin America, one on North America, four on Asia and four on Africa. This regional bias partially comes back in the illustrations throughout the paper. However, the objective is not to provide a representation of all existing RCA-related formats and experiences. Rather, we chose to include illustrations that are based on long-term involvement (as researchers, activists or both), which allows to write from a substantiated understanding rather than a fabricated representability. In a similar manner, we believe that they are sufficiently diverse to provide a contribution and orientation (not a blueprint) for those interested in RCAs. Lastly, it is important to clarify that most of the authors define themselves as activist scholars, meaning that we take an explicit political standpoint in our work and engage with groups that have been disadvantaged and struggle for reviving rivers and riverine livelihoods. This activist position calls for openness, solidarity, and forms of scientific rigour that preserve the integrity and agency of all those involved, while facilitating reflexivity, deep co-learning, and the creation of actionable knowledge. Such a political stance clearly influences our work; it was the main motivation for engaging with RCAs in the first place.

In the following, we first conceptualise and clarify our understanding of rivers as complex networked and relational spaces, in order to set the stage for both the RCAs themselves and our related contemplations. Subsequently, in Section 3, we have sought to situate RCAs in the context of scholar-activism, and to articulate the fundamental relationship between this approach and the engagement of academia with social movements advocated here. Next, in Section 4, we bring the aforementioned reflections in conversation with relevant theories and concepts, and sketch cornerstones for reflexive enaction of RCAs and the related learning approaches and practices. By way of conclusion, we critically ponder on the approach presented here.

## Understanding riverine complexities

In theory, RCAs can be places, platforms, networks, and encounters that invite and support shared learning, reflection, creation, and action. Independent from their place – and time-specific shapes and objectives, they necessarily evolve from and take place within socio-ecological riverine systems that entwine human and non-human lifeworlds. In order to understand this complexity of the riverine context from which RCAs and associated NWJMs emerge and within which they act, it is useful to consider rivers in terms of interconnected dimensions or ontologies. The starting point is an understanding of rivers as networked relational spaces that are simultaneously material, technical, social, and symbolic (Bakker 2012; Swyngedouw 2015). Elaborating on Boelens et al. (2023), we deploy four inter-connected ontological fields or windows to explore this understanding.

First, “river” refers not to flows of H2O but to the socio-ecological relations and entwinements that co-produce *rivers as ecosociety* – as socionatures – constituted by the interplay of hydrology, ecology, climates, landscapes, and societal cultures, institutions, technologies and politics on multiple scales. In other words, river basins, wetlands, streams, and hydrological cycles are mediated by physical-material elements, hydrological models, and technological interventions, as well as by human imaginaries, institutions, and relationships (Ibid. Cf. Buijse et al. 2002; de Jong et al. 2024; Harris and Heathwaite 2012; Krause 2013; Wantzen et al. 2016).

Second, this also implies that it is relevant to consider how actors may have different, overlapping or contrasting visions about what the socio-ecological river is and how the relations between and among human and non-human communities should be ordered (Houart, Hoogesteger, and Boelens 2024). These visions are often subject to negotiations and contestations between riverine actors, who each try to enact and materialise their visions and respective interests in rules, institutions, river infrastructures, and access-distribution patterns. Power is central in these processes that create *rivers as territories*, and can range from overt force, laws, and policies to discourses, narratives, imaginaries, and subject formation (Boelens et al. 2016; Hommes and Boelens 2018; Krause 2017).

Third, different political arenas and cultural framings constitute and promote socio-ecological *rivers as subjects*, not just objects, to enact and uphold hydrosocial relations. This applies to both the river’s human communities (e,g, the creation of specific water users, water “managers” or river guardian subjects) as well as to the non-human/ecological communities, sometimes recognised as legal or political subjects through, for instance, Rights of Nature notions, policy proposals or citizens movements’ initiatives (Latour et al. 2018; O’Donnell and Talbot-Jones 2018; Chaturvedi 2019; Tănăsescu 2022; Cohen et al. 2023; Houart, Hoogesteger, and Boelens 2024; McNeish and Socha 2024; on more-than-human riverine relations, also see e.g. Behn and Bakker 2019; Erwin et al. 2022; Hikuroa et al. 2021; Reyes Escate, Hoogesteger, and Boelens 2022; Smith 2012; Vinyeta, Whyte, and Lynn 2015; Wilson et al. 2019). Important is to consider how people and rivers are politically made into specific subjects and with what political, cultural, legal, and moral impact (Boelens et al. 2021): how they are subjected as subjects (Foucault 1995) or, alternatively, how they may claim voice and self-determined rights as agents and subjects (and what role RCAs can play in this context).

Finally, rivers move, catalyse, and inspire people and actions: *rivers as movements*. RCAs are an intrinsic part and manifestation of this dimension as they are the relations and place around which movements mobilise for claiming water justice and transformative co-governance of river commons. At the same time, while rivers are the central focus and place for NWJMs and RCAs, many river movements operate on multiple scales and act on multiple issues at the same time (Boelens et al. 2023). Just as the river as ecosociety is shaped by local, regional, and global dynamics alike, so may movements engage with manifold scales through vertical networking, e-activism or embedding their struggles in globally operating networks or discourses much beyond managerial river scales such as the “river basin”.

RCAs are embedded and take shape in this riverine complexity that we tried to conceptually disentangle in this section. The idea of socio-ecological co-production (“river-as-ecosociety”) is, in fact, deeply entrenched in the idea of RCAs as knowledge and action co-creation arenas, and leads to an explicit engagement with the human and non-human constituents and their interrelations. The second ontology (“river-as-territory”) calls us to pay attention to power processes at play in the negotiations around river relations and to the existence of multiple contested visions of what a river is and should be. Considering “river-as-subject” positions RCAs as an opportunity to reflect on how subject identities are experienced and claimed, and to devise actions for self-determined subject claims. Last, RCAs are among the many expressions of “river-as-movement”, as they mobilise diverse actors to co-produce knowledge, engage in dialogue and learning, bridge diverse contexts, and hybridise and elaborate river enlivening proposals that pose alternatives to solely technocratic-hydraulic development strategies.

Ultimately, the outlined ontologies stand for ways of understanding and engaging with river systems in different contexts, cultures, and time-scales. In the context of RCAs, they can open up new ways of understanding, interacting with and mobilising around the dimensions that rivers entail and bring together. These ontologies interweave and complement one another and lead to questions that provoke reflections on current river’s socio-nature configurations and future orientations that are to be encouraged and actively mobilised within RCAs operations.

## RCAs in the context of activist research

In the above sections we have outlined the complex contexts in which RCAs are likely to operate, and underlined their embeddedness within the multifaceted landscape of social mobilisations aimed at addressing river-related problems. Studies on social mobilisations as in India (e.g. Khagram 2004; Cortesi and Joy 2021; Shah et al. 2021), Spain (e.g. Hernández-Mora et al. 2015; Sanchis-Ibor, Boelens, and García-Mollá 2017; Villamayor-Tomas and García-López 2018; Prieto López, Duarte-Abadía, and Boelens 2021), Ecuador (e.g. Bebbington and Perreault 1999; Goodwin 2019; Hidalgo-Bastidas and Boelens 2019), Iraq (Rizzi and Mollinga 2024) and Peru (e.g. Bebbington, Bebbington, and Bury 2010; Heikkinen 2024; Hoogesteger and Verzijl 2015) have shown that the strength of these mobilisations depends on the capacity to integrate diverse scales and include different forms of knowledge and multiple actors, with different roles (cf. Cumbers, Routledge, and Nativel 2008; Oslender 2016). Such integration and inclusion allows the implementation of manifold actions at different scales and the formation of coalitions to advance the movements’ interests, disseminate their discourse, make their claims heard, and contest dominant power structures (Suhardiman, Nicol, and Mapedza 2017; Van den Berge, Vos, and Boelens 2022).

In this context, the solidarity partnership between social movements and activist scholars through action research practices has proved fruitful. At the heart of activist research approaches is an emphasis on the deconstruction of academic and policy practices that universalise, normalise, and create hierarchies of knowledge, in favour of a reconstruction that embraces plurality and counteracts colonialism and all forms of oppression and domination (Fals-Borda 1987; Damonte and García 2015; Routledge and Driscoll Derickson 2015).

Lately, a greater number of settings – academic institutions, civil society organisations, governments, and communities – have enabled modes of participatory research in which scholarship commits to socio-ecological justice and democratisation of knowledge, fostering collaboration between academia and activism (Sangameswaran, Narain, and Joy 2013). Funding agencies, governmental arenas, and other opportunities have been broadened to enable more meaningful co-learning and engagement to focus on environmental justice. As a result, a range of participatory methods for knowledge production has emerged over time, from participatory action research to community-based participatory research, participatory rural appraisal, participatory modelling, photovoice methods, citizen science, sustainable futures scenario building, science policy stakeholder interaction, among others (e.g. Castleden and Garvin 2008; Tremblay and Harris 2018). Beyond this, the critical deployment of decolonising research methodologies that strive to centralise indigenous, vernacular and grassroots knowledges and perspectives, in order to unravel colonial legacies and power dynamics embedded in academic research has gained traction (Mignolo 2011; Smith 2012; Koppes 2022; Lang et al. 2012). In the context of water, there are numerous inspiring examples, from work centred on gender diversity and women's roles in maintaining waterways and riverine environments (Vinyeta, Whyte, and Lynn 2015), to highlighting relationships of care (Daigle 2018), or expanding knowledge pathways around storytelling and familial and everyday relationships (Craft and King 2021).

These advances in decolonising and transdisciplinary approaches form a fertile ground for the conception and implementation of RCAs. RCAs provide a broad platform for enriching and opening a much wider canvas for interactions between researchers, activists, community groups and social movements, and between knowledge and politics. However, this critical engagement is far from easy. There are many barriers to overcome, including the hierarchies and boundaries of knowledge, the underlying identity and political/conceptual positionality of the scholar/researcher and the activist, and the pervasive scepticism among activists and movements about academics and their usefulness for change.

Either way, activist research undertaken through the RCA will inevitably navigate tensions posed by a “dual loyalty”: both to academia and the production of rigorous academic analysis (which involves a critical perspective to the broader societal inequalities as well as to local, internal injustices in river commoning initiatives), and to the practices and political struggles of the groups with which it is engaged (Hale 2006; Suhardiman and Middleton 2020). In RCA practice, it means finding the themes of research in partnership with people who are embedded in and experiencing daily the local river-related issues, building a joint knowledge project. As Hale (2006, 98) notes, this rather complex position of the activist scholar means occupying “a space of profound generative scholarly understanding but it also means entering into the compromised conditions of the political process” at stake. For academia, it holds considerable potential to yield new insights to conventional scholarly knowledge (being judged for its analytical rigor); outside of academia, the research may offer a possibility to analyse the contradictions, languages, and practices used by the groups in struggle (being judged for its concrete contribution to the local political struggle with which it engages) (Hale 2006). Ultimately, the realisation of the transformative potential of RCAs in this context lies very much in its ability to effectively answer the question of how much activist scholars actually co-produce knowledge for transformative practices and how much social movements can benefit from scholar-activism (Joy 2021).

## Collectively identifying, challenging, and producing river knowledge through RCAs

After having engaged with how to understand RCAs in the context of river struggles and how they are situated in the realm of scholar activism, we now want to turn the attention to cornerstones that we consider crucial for organising RCAs or engaging with them. First, we position the challenging of dominant truth regimes and the co-creation of “counter truths” as one of the cornerstones for RCAs. Second, we argue that RCAs build on transgressive co-learning processes that precisely defy such truth regimes and make room for the emergence and appreciation of alternative ways of thinking (e.g. river not just as resource for hydroelectricity but as life giving for multiple species). As we argue later, such co-learning processes and the interaction of multiple and diverse actors within RCAs inherently navigate dynamics of collaboration and confrontation, which can be leveraged for productive outcomes. We then discuss “ongoing reflexivity” as a way of recognising intersecting subjectivities, power dynamics, and anchoring RCA processes. Finally, we consider the potential for exchange of experiences and knowledge between multiple RCAs to advance understandings and practices around river struggles.

### River knowledge encounters and truth regimes

Rivers as ecosociety, territory, subject and movement are inherently produced and ordered by power processes and entwined with specific ontologies that are validated (or de-valued) by particular epistemologies (modes of knowing, or “truth-regimes”) and that actively produce river realities with concrete social, material, and symbolic results (Behn and Bakker 2019; Boelens, Shah, and Bruins 2019; Duarte-Abadía, Boelens, and Buitrago 2021; Jaramillo and Carmona 2022; Götz and Middleton 2020). In fact, knowledge and power inherently depend on each other. In river governance contexts, thus, the workings of power produce river knowledge, river truth claims, river facts. As Foucault (1980, 133) argued, “truth is linked in a circular relation with systems of power which produce and sustain it, and to effects of power which it induces and which extend it, a “regime of truth”. For instance, one of the dominant knowledge production paradigms in river management is the use of numerical models, such as for simulating and evaluating water quality and water quantity measures. The land-use maps that underly many models are (often implicitly) politically constructed (Comber, Fisher, and Wadsworth 2002). As such, models conceal certain values that have materialised in the model code, thereby favouring certain views at the expense of others (Melsen, Vos, and Boelens 2018).

A major objective of RCAs, therefore, is to critically examine dominant (State/market/science) river knowledge production, and the way it orders river knowledge and “knowers” according to its own degrees of validity, subtly separating “legitimate” and “truthful” from “mistaken” or “illegitimate”. As Vos and Boelens (2014) show, this occurs commonly by deploying depoliticised but deeply normative criteria regarding, for instance, appropriate hydraulics science, notions of efficient and productive water use, equitable and rational water allocation, modern water users, smart and sustainable river development. RCAs can play an important role to challenge such dominant truth regimes and co-create “counter-truths” and alternative configurations.

However, contesting dominant truth regimes and creating other truths is challenging; especially so because modern (e.g. legal, scientific and/or neoliberal-market) normalisation procedures are not solely based on top-down control, exclusion, and discrimination but on productive values, including knowledge inclusion and participation (Boelens, Shah, and Bruins 2019). Numerous examples can be found along this vein. de Castro (2012), for instance, examines how the early participation of local residents in designing a territorial model for the Lower Amazon Floodplain resulted in the legal recognition of a community-based fishing territory but how, little after, a constrained timeframe, technocratic methods, and a history of patronage turned its implementation into a rushed, top-down process with minimal sustained local participation. As a result, local tensions exacerbated and prevailing power dynamics remained unchanged, rather than fostering socio-environmental justice.

Even more so, the most powerful norms are those that do not appear to come from “outsiders” (e.g. “the West”, the “colonisers”) or “rulers” (“elites”, the “government”), but from “within” (internalised norms), as part of the implicit and interiorised perceptions separating what is marked as “normal” from “abnormal”. As a result of this dominant politico-economic and discursive ordering power, the plurality of existing knowledges, ontologies, skills, meanings, values, and rights related to vernacular, indigenous, place-based understandings and orderings of riverine territories becomes subordinated (cf. Feindt and Oels 2005; Foucault 2008; Dukpa, Joshi, and Boelens 2018; Oslender 2021). Therefore, one important step for the RCAs is to collectively identify these repertoires of “subjugated” as well as “dominating knowledges” (Foucault 1980, 82), and understand how some knowledges are disqualified, made hierarchically subordinated, and/or appropriated into dominant discourses without attribution, while others are elevated to the required levels of legal or scientific recognition (Hale 2006; Harris 2017; Illich 1981, 1986).

Thus, RCAs need to scrutinise knowledge encounters, dissect processes of knowledge subjugation and how they happen through selection, normalisation, hierarchisation, and centralisation (Foucault 2003), and engage in riverine battles for truth. Clearly, this is not a neutral and objectifying science battle on behalf of a singular truth, but a transcultural, transdisciplinary and grass-rooted battle “ … about the status of truth and the economic and political role it plays” (Foucault 1980, 132). Box 1 illustrates this in practice: how alternative methods such as storytelling and memory sharing can be employed as powerful means of challenging dominant truths while promoting counter-truths, honouring generative and life-enhancing ways of living with rivers. The project was conducted by the collaborator team of one of the authors, over several years, in the north-eastern region of British Columbia. It demonstrates the contrasting perspectives between dominant discourses about the river as a resource to be exploited and indigenous cosmologies, which affirm the river's intrinsic value.

Box 1. **Working towards alternative visions and thinking natures and rivers “otherwise”.**

Several examples allow us to interrogate truth claims about rivers and how they might be overtly challenged and contested. Drawing on examples from North America, while state and engineering efforts of the past century dammed and diverted many rivers for hydro-electricity generation and other developmental goals, we can observe direct challenge to the ideas of rivers as “resources”, instead focusing on rivers as “lifeblood”, kin, or the veins of Mother Earth. Such visions and understandings are often more in line with indigenous cosmologies, ontologies, and epistemologies (Daigle 2018; Wilson et al. 2019). Bringing in alternative ontologies and epistemologies can help us think and manage rivers “otherwise”. An analysis of conventional approaches to assessing dam infrastructure, focused on the Site C dam on the Saaghii Naachii/ Peach River in northeastern British Columbia (BC), Canada, Caleb Behn and Karen Bakker (2019) offer an alternative assessment informed by Dunne-Za teachings focused on the interrelationships between land, water and animals in the river basin. They show, among other things, that plural and indigenous approaches to assessing the dam and its impacts leads to radically different understandings of the stakes of such developments. With storytelling and memories of hunting and living along the river shared by Behn, juxtaposed against more conventional scientific assessment, the work highlights the types of knowledges (and formats) that are often valued, as well as those that are excluded, opening up spaces to reflect on more “expansive, pluralistic” approaches in line with the theory and potential of RCAs. Engaging more directly with the politics and knowledge associated with Community Based Monitoring (CBM), analyses have also explored the ways that diverse CBM practices have too often been carried out in ways that reinforce, rather than challenge, Western, colonial, and capitalist systems and economies (Cohen et al. 2021).

### Transgressive co-learning

Playing out transcultural, transdisciplinary, grassroots battles that challenge truth regimes through RCA requires contravening what is normalised and opening spaces to allow subjugated river knowledges to be heard. This implies collective learning processes that are set in motion by RCA participants in order to engage with and develop new understandings of river as ecosociety, territory, subject, and movement towards co-producing alternative river realities. Transgressive co-learning processes are therefore at the basis of RCA practices. Transgressive co-learning seeks to weaken the rigidity inherent in established structures, challenge conventional approaches, and disrupt entrenched dependencies on unsustainable patterns, thereby catalysing the emergence of alternative pathways. The immediate aim is to create disruption, while the ultimate aspiration is to bring about a major redesign or overhaul of the system based on new principles, ideas, and values. The prefix “co” in the context of co-learning is critical, as it emphasises the gathering of voices and knowledges that can challenge prevailing norms and defy conventional paradigms.

Underpinning these processes is a *relational* understanding of the transgressive co-learning practices within RCAs: embracing a type of learning that is inherently dependent on the interactions of the actors involved and on one's own sensory and practical experience of the environment in which one is embedded (Huijbens 2021; Souza, Wals, and Jacobi 2019). Incorporating such a relational perspective requires an immersive exercise in which one attends to all the relationships that animate the context, including actors’ own role in the unfolding of the events (Escobar 2016).

Crucially, this approach seeks to act at the very interface between nature and society, avoiding any sort of dualistic thinking. Therefore, while this approach to RCAs celebrates plurality, immersive experiences, and ongoing unfolding, it avoids a debilitating relativism by reflexively engaging with the goals and ambitions of the RCA in each setting (this point will be further elaborated later, in reflexivity within RCAs). Adopting such a relational stance is a rather radical departure from traditional empirical approaches to knowledge construction, which assume that what is to be known is simply out there, readily representable. Privileging the experience of what is to be found “out there”, the classical empiricism, not only runs the risk of reifying (no matter how elaborately) what is to be encountered but also tends to overlook assumptions of the ones involved in knowledge production and ways of constructing and adding to their mode of engagement and knowing.

Thus, next to the more common natural and social sciences methods for knowledge construction, alternative practices that are highly experiential and integrate sensing, feeling, and thinking are suited within RCA activities (cf. Scheffer and van Nes 2018). This might include arts-based approaches as a way of tackling and fostering new views on problems (Boeckel 2019), play as a form of dealing with issues that can be disruptive (Eernstman et al. 2021), or theatre to reimagine possibilities and give new meaning to seemingly hopeless situations (Erwin et al. 2022). Practices such as concern-driven citizen science or so-called civic science (Dillon et al. 2016), also have the potential to integrate experiential, hands-on field research with scientific assessment, promoting deeper levels of learning. At the same time, as Turnhout (2022, 6) warns us, “citizen science makes clear that participation in science and technology is challenging, and that it can often, despite good intentions, produce negative outcomes”. For instance, “processes that simply invite all perspectives and stakeholders to the table without addressing power often result in the strengthening of existing power inequalities” (for illustrations, see e.g. Duarte-Abadía, Boelens, and Buitrago 2021; Dukpa, Joshi, and Boelens 2018; Dupuits et al. 2020; Jaramillo and Carmona 2022), or, as commonly happens, “processes that prioritise consensus or integration often end up excluding those voices that are seen as unreasonable and uncooperative” (Turnhout 2022, 6). Oslender (2021) in turn highlights how, if critical power issues are addressed, the transformation of interiorised perceptions and imaginaries can be catalysed. Addressing these issues means both opening up spaces for historically oppressed voices to be heard and acknowledged, as well as inviting a wider and diversified range of views into the conversations. Oslender (2021), Hommes, Hoogesteger, and Boelens (2022) and Vos (2024) stress experiences brought about by protest marches to restore river health, community river clean-up campaigns, or diagnostic walks, and collective counter-map making as engaging activities that can be highly conducive to transforming visions and practices. These different methods and formats all fundamentally involve dialogue as central to the collective learning processes advocated here. However, the kind of critical dialogical interaction that can be achieved through RCA processes is crucially not simply a consensus-seeking conversation, as we will further develop in the next subsection.

Box 2 shows an example of counter-mapping processes in Colombian and Ecuadorian rivers to induce transgressive co-learning experiences, in which three of the authors of this paper were involved along with several PhD researchers. These workshops, which took place in 2023, were one of the first RCA experiences put into practice by the Riverhood and River Commons projects and the Travelling Rivers initiative along with fishing and indigenous communities who claim to maintain their generative practices and ways of living and coexisting with rivers. This initiative not only promoted the making of the counter-maps, but also used them as elements to bring the communities of the different rivers involved into conversation. The main question posed by this transdisciplinary activist research initiative was: How does the interconnection and interweaving of different socio-fluvial representations and contexts (notions and discussions) generate new understandings and concepts capable of transforming hydro-social realities? The aim was to illustrate, mobilise, and synergise the hidden river knowledge, imaginaries, and aspirations through the mapping of grassroots realities and collective strategies for the revitalisation of rivers.

Box 2. **Transgressive co-learning experiences through counter-mapping.**

Mapmaking has historically been one of the principal tools employed by dominant powers to appropriate territories for utilitarian purposes. Counter-mapping challenges this traditional standard of empiricism that decentres communities and focuses on a narrow set of voices and insights, and provides a new approach to co-creating and generating knowledge – key facets of transgressive learning. It can shed light on dominant power relations and the embedded contested practices, whilst providing a space for critical, creative, and inclusive reflections. By utilising the porosity between people and places that is silenced in traditional map-making processes, it encourages transgressive learning. A series of counter-mapping workshops that were held on six rivers in Colombia and Ecuador in 2023 are an example of this. These workshops were directly led by grassroots artists in collaboration with researchers and local riverine communities affected by various activities impacting their rivers, such as mining, dam construction, agro-industrial pollution, and weak local leadership of community irrigation systems. The counter-mapping method used by the artists leading the process was based on their extensive experience of working with communities, combining the artists’ skills and practices with the communities’ previously acquired mapping skills, making it easier to approach and integrate the different local voices present. These workshops provided a platform for these communities to articulate their sidelined perceptions and lived experiences. This inclusive process enabled a deeper understanding of the disruptive forces affecting river-based livelihoods, the convergence of divergent viewpoints, strengthening of local and trans-local coalitions to address place-based challenges and the emergence of alternative narratives in line with local values. This endeavour served to affirm the legitimacy of local knowledge systems and practices, and to challenge entrenched power structures. It transcended conventional empiricism, by promoting a holistic and inclusive approach to envisioning a wide range of positive engagements with river systems. The grassroots artists further acted as “river connectors”, taking maps, stories and “video letters” from one river site to the next, fostering debate, solidarity and learning across local river contexts.

### Confrontation and collaboration dynamics

RCAs provide an interactive space where various actors come together to scrutinise, expose, and produce alternative river knowledge through dialogue, critical reflection, and trust-based relationships (Davidson-Hunt and O’Flaherty 2007). However, in the complexity of river contexts and at the interface of river as eco-society, territory, subject, and movement, the interactions between the various actors involved are far from harmonious processes in which everyone can easily agree on opinions, ideas, positions or interests.

Critical to transgressive co-learning fostered by RCAs is an understanding of how power dynamics, both implicit and explicit, shape the interactions between the various RCA participants, and how these dynamics intersect with the production of alternative river knowledge. Ideally, RCAs provide possibilities to express opposing and contested viewpoints among those in interaction while striving to build alliances and fostering the power of collective and individual agency to claim rights by recognising and valuing rooted epistemological and ontological pluralities (see Vos et al. 2020)

Thus, while actors come together under a common – temporary or permanent – interest, hope or larger political project they aim to advance (e.g. Boelens, Bustamante, and Perreault 2010; Hoogesteger 2013), the co-creation of knowledge and action between different actors also always encompasses a confrontation of values, discourses, norms, epistemologies, and ontologies. Therefore, it is vital to recognise these dynamics of conflict and confrontation as an inherent aspect of collaborative efforts within the RCA itself, and between actors involved in the RCA and external ones (Harris and Alatout 2010; Zwarteveen and Boelens 2014; Roca-Servat and Ocando 2019; Joy et al. 2020).

Importantly, these conflicts and conflicting perspectives cannot and should not be “resolved or facilitated away” in search of rational and participatory consensus, but rather harnessed and turned into the backbone for disrupting practices. This means tapping into the transformative potential of political contention within RCAs, in line with the principles of “agonistic pluralism” (Connolly 2013; Colloredo-Mansfeld 2009; Mouffe 1999). In agonistic pluralism, opponents converge to articulate their political differences over meaning and power, searching for new perspectives that challenge claims of “established knowledge”, “authoritative rules”, or “rational, participatory and deliberative consensus”. In this context, dialogic interaction taking place within RCAs provides the fertile ground for nurturing agonistic frameworks.

In dialogical interactions the views expressed by RCA participants may not be observed as neutral truth claims that can be objectively accessed, but as constructions and interpretations of the world subject to transformation. In practice, dialogical encounters among RCA participants would require (1) maintaining an open stance towards recognising and respecting ontological pluralism and silencing pre-assumptions so to cultivate a distinctive quality of listening and openness to constructive exchanges, (2) the use of self-reflexive, self-critical, supportive facilitation (Jiggins, Roling, and van Slobbe 2009), and (3) continuous collective reflection on the litmus test question: “who gains and who loses”, how are benefits and burdens socially distributed when adopting, deploying or constructing particular river ontology and knowledge claims (Schlosberg 2004; Cumbers, Routledge, and Nativel 2008; Perreault, Bridge, and james 2015; Harris 2017). This means that RCAs are spaces where agreements will be not easily reached but where plural views can potentially be included in agonistic configurations to promote alternative river knowledge (Hillman 2004; Wals, van der Hoeven, and Blanken 2009). Similarly, it is necessary to acknowledge that the confrontation between groups with opposing views may prove unproductive as a result of an inherent impossibility of achieving negotiation between disparate ontological spheres and/or due to the power dynamics that may be at play. Moreover, it may be impractical to guarantee the inclusion of all individuals related to the subject matter within the RCA dialogical field. It is therefore vital to recognise and critically reflect on the inherent limitations of the real possibilities of an RCA and how this is reflected in the actions generated from it. The dynamically changing responses to the core questions of “whose knowledge prevails” and “who wins, who loses” need constant reflectivity (Duarte-Abadía, Boelens, and du Pré 2019, 2021; Dupuits et al. 2020; Turnhout et al. 2020; Zwarteveen and Boelens 2014). It is of particular importance here to note that the entirety of the operation is based on the premise of partial results and an ongoing process of action.

At the level of collaboration and confrontation with actors outside of the RCAs it is as important to identify what institutional, political-strategic, and cultural-symbolic relations exist and how the participants of the RCAs (may) relate to these (e.g. Cumbers, Routledge, and Nativel 2008; Gerlak et al. 2011; Hoogesteger, Boelens, and Baud 2016; Manosalvas, Hoogesteger, and Boelens 2021). In this sense, fundamental questions need to be raised with regards to the following issues: What, how, and why do actors define “inside” and “outside”, “internal” and “external”, considering that distances are relational. What is the purpose of collaboration or confrontation with external actors? What strategies can be used to engage with external actors? What are the risks and opportunities of these engagements? How can such engagements be organised and strategised and what is needed for it? These are issues that we consider have to be explicitly addressed in the context of RCAs and need to be constantly evaluated throughout the development and/or support and participation of and in these initiatives.

Box 3 illustrates a dynamic of collaboration and confrontation in a multi-stakeholder coalition in the Santurban Páramo, Colombia. This coalition was formed to defend the conservation of the site and the well-being of the communities that inhabit it in the face of the threats posed by mining activities. However, this joint effort revealed internal conflicts that required the creation of new platforms for dialogue in order to resolve tensions and advance the protection of the páramos. One of the authors of this article actively participated in these dialogues between 2011–2015 and 2018–2019. The research aimed to understand how different actors hold different values and perceptions of the páramo, connected to different approaches to manage and use it. In addition, the study analysed how rural-urban water transfers restructure hydrosocial territories in line with neoliberal ideologies, raising environmental (in)justice concerns.

Box 3. **The tides of confrontation and collaboration in the case of Santurban paramo in Colombia.**

At the beginning of the 21st century, with the expansion of the extractivist model, many of the Andean highland wetlands were increasingly titled for mining exploitation. One such case is the Santurban paramo in the northeastern Colombian Andes, where several multinational companies have attempted to extract gold on a large scale, threatening the water supply of the city of Bucaramanga. In response, civil society groups in Bucaramanga organised the “Committee for the Defence of Santurban Water and Páramo”, in 2000. The Committee comprised an extensive and heterogeneous network of around 40 organisations, including student movements, the Bucaramanga Metropolitan Waterworks, NGOs, associations of entrepreneurs and industrialists, political parties, grassroots communities, international organisations and academics (Duarte-Abadía, Boelens, and Buitrago 2021). In an effort to resolve the conflict and “balance the different interests” between the Committee and the mining sector, in 2014 the Colombian government demarcated and zoned the paramo for restoration, sustainable agriculture and conservation. Within the “restoration areas”, mining activities could continue if the mining titles were obtained before 2010. However, this joint strategy by the government and the transnational company to manage and incorporate the differences was legally rejected by the Committee in 2015, as it violated the right to environmental participation (the affected population was not informed and consulted during the delimitation process). Meanwhile, tensions between the people of the paramos and the Committee have quietly escalated. For the Committee, peasant livelihoods based on agricultural production, combined livestock rearing and artisanal gold mining have led to the depletion of the paramos, affecting water quality and supply. The peasant families, on their turn, disagreed with the topdown and (market-)environmentalist conservation proposals and impositions (Duarte-Abadía, Boelens, and Buitrago 2021). As a result of these urban-rural tensions, the Collective of Peasants and Community Reserves of Surata began to create spaces for dialogue with grassroots communities in Bucaramanga, such as peasant markets and cultural festivals (Roa-Avendaño 2024). These meetings have changed the vision and environmental discourse of the Committee to protect the paramo. They now recognise the cultural importance of defending their rivers and water sources together with the inhabitants of the paramos.

### Ongoing reflexivity

As we have mentioned, active and ongoing individual and collective reflexivity in relation to the interactions that take place within RCAs is crucial. This may involve critical scrutiny of the intersection of political standpoints, positions of power and social markers (e.g. class, gender, race, ethnicity, age and others) that shape experiences and possible disadvantages of actors interacting within and outside RCAs.

As highlighted in section 3, academia, and more specifically the activist researcher, occupies a significant position in our discussion of RCAs. The researcher is positioned as an engaged figure and/or catalyst within the processes and practices of RCAs, working with multiple actors to co-produce knowledge for action. As such, the researcher emerges as a political actor capable of facilitating connections between local actors, as well as other contexts and individuals. Researchers can even link various geographically disparate RCAs to exchange experiences and form translocal networks (see Box 2 and next section). All these translations and connections imply interactions that affect the context and relationships with participants, inside and outside RCAs. Thus, the researcher's reflexive stance on their motivations, interests, attitudes, forms of involvement and actions, and the resulting consequence and influence on and within RCA practices and on the movements and people with whom they relate, is a constant challenge throughout the process of engagement (Roca-Servat and Ocando 2019; Veldwisch et al. 2019; Joy et al. 2020; Hidalgo-Bastidas 2020; Duarte-Abadía 2022). Also, activist scholarly engagement with riverine communities may require attention to communities’ relational approach to rivers (where applicable), which implies a departure from the prevailing anthropocentric perspectives pervasive in academic and policy discourse.

In order to meet these challenges, however, it seems insufficient to just consider the positionality of the researcher within the scope of an “anticipatory reflexivity” or “transparent reflexivity” in which points of view and stances are specified at the outset, independent of the research process (Arias López, Andrä, and de Guevara 2023). Within the evolving interactions within an RCA, positionalities and perceptions are likely to change. New subjectivities may emerge through multiple interplays and exposure to alternative perspectives, dissonance, disorienting dilemmas, and questioning what is normal. Speaking of “continuous” or “relational” reflexivity in the context of a fluid process in which cultural, political, institutional processes and structures are at the background of the RCA encounter (Nagar and Geiger 2007) and in which multiple and emergent identities need to be observed and analysed, therefore seems more appropriate (Soedirgo and Glas 2020).

Beyond the researcher, continuous reflexivity must also extend to others involved in the process (i.e. group of researchers, co-researchers, individuals involved in RCA) through collective reflexivity dynamics. In this form of reflexivity, the involvement of the group can serve to bring to light facets obscured by solely an individual introspection. Collective reflexivity may not only broaden the scope of individual reflexivity but also facilitate the emergence of alternative perspectives (Arias López, Andrä, and de Guevara 2023). Such reflexivity can be nurtured among all those involved in RCA to enhance the recognition of the multiplicity of viewpoints within the collective, while potentially fostering a sense of shared support among participants (Arias López, Andrä, and de Guevara 2023). This can be crucial for navigating the uncertainty that arises from practices that deconstruct established narratives and construct new ones.

Finally, within the scope of ongoing reflexivity, it is essential to acknowledge the inherent limitations of this practice. A comprehensive understanding of the myriad of factors at play, as well as the intricate dynamics of power and its ramifications, may prove to be elusive. What emerges as a result is a form of reflexivity that is by its very nature contingent, self-critical and, in this respect, radical (Maxey 1999).

Box 4 illustrates two projects in the Cauca and Magdalena Rivers, in Colombia, and how engagement with riverside communities and its environment can affect the researcher's sensibilities and ways of perceiving rivers. The projects presented in the table involved two authors of this article.

Box 4. **Shifting perspectives on rivers as ecosociety through reflexivity in action research in Colombia.**

Through continuous reflection – individual and collective – researchers can gain insight into the multifaceted dialogue between riverine communities and their surrounding rivers, such as in the projects “Water Security Hub in the upper basin of the Cauca River” (initiated in 2019) and “Thinking with Rivers about Environmental Peace in Colombia” (started in 2023 along the Magdalena River). Involved communities have revealed the reciprocal ties they have with the rivers, as exemplified by the inhabitants of the Sonso lagoon, who claim that “the river warns us when it is going to rise, and we retreat”. Similarly, the leaders of Quinamayó underline the agency of rivers by stating that “the river has left us a 15-hectare plot of land, and we decide collectively what to do with it”. In working with riverine communities, researchers can become aware of their own estrangement from nature and come to see it through a different lens. They may appreciate previously imperceptible sounds, tastes, smells, and colours. The reciprocal interactions between nature and communities defy simple comprehension within modernist-scientific and techno-political frameworks that overly dissect reality into isolated components in order to understand how it works. Moreover, this immersion in the field reveals the political tensions that rivers and communities are caught up in as they strive to preserve livelihoods that are threatened by exploitative economic interests that fail to recognise such innate reciprocal human-river relationships. Through a reflexive examination of the researchers’ roles within this politically charged context, new political stances, positionalities, and perceptions emerge. In the case of the two projects, artistic expressions were integrated into the ongoing action research in order to harness and express the potential of alternative ways of interacting with reality (e.g. through painting, collage, or drawing), thereby also aiding the researcher's reflexive process.

### Transcultural knowledge assemblages and translocal bridging of rooted knowledges

While RCAs are likely to be initially place-based and rooted in a specific hydrosocial and geographic context, they can also be articulated through practices and in assemblages that cut across spatial boundaries. RCAs established around river cases in several continents, despite being configured as entities that will differ according to the cultural reality that shapes them, are likely to share aspirations and goals related to the deployment of alternative grassroots river governance practices and co-production of new river knowledge. Transcultural knowledge assemblages can therefore emerge from connections between heterogeneous RCAs (see Box 5). Identifying converging themes, concerns, interests, and practices that define interfaces capable of linking the different riverine practices becomes fundamental to enact such assemblages across spatial boundaries.

Transcultural knowledge assemblages emerge through networks that are fluid, decentred, temporary, horizontal (Cumbers, Routledge, and Nativel 2008), formed not only between diverse RCAs, but also between RCAs and other actors (e.g. NGOs, trade unions, social movements, activist academic groups). They are also a political process that not only requires engagement with diverse actors and interests in particular places and across scales, but also a negotiation that “inevitably entails contestation and an ongoing consideration of diverse options and trade-offs” (Harris, Chu, and Ziervogel 2018, 196).

Such articulation among RCAs will require translation processes that bridge convergent rooted river knowledge (Vos et al. 2020) while respecting the differences and peculiarities defined by the RCAs’ fixity in one place (Kinkaid 2019). In this process, Cumbers, Routledge, and Nativel (2008) emphasise the concept of “brokerage”: a set of actors, among which possibly researchers, who have the capacity to bridge different networks, communities and RCAs with converging themes. Practical forms of RCA bridging and translocal network formation can be virtual and/or material, including internet, open publishing, alternative media sites, place-based events, union meetings, among others. Among the many possible modes and constellations, live gathering is fundamental for creating trust and mutual solidarity, and thereby increasing the chances of establishing sustained translocal interactions (Cumbers, Routledge, and Nativel 2008). Also, different material resources, mobilisation capacities, and inequalities between river movements must be taken into consideration as they define different potentials for building transcultural knowledge assemblages. It is worth reiterating the experience reported in Box 2, which exemplifies the potential of counter-maps as a tool for connecting multiple movements. The initiative in question employs maps as a means of making the struggles for river regeneration visible, functioning as a communication vehicle that facilitates dialogue between various actors, thereby supporting co-learning and co-production of knowledge.

Box 5 showcases how civil society organisations serve as institutional bridges interconnecting scattered communities to form transcultural assemblages in the Mekong region. The converging issue in this case was the struggle against hydropower dam development. It focuses on how local communities along the river could build strategic alliances to counter force the dominant power relations that favour and impose dam development on communities’ livelihoods. The collaborative production of impact assessments led by two civil society organisations together with local communities constituted the means through which information was shared and local movements linked and strengthened, despite the countervailing forces of the dam construction company. This data is based on one of the author’s field research in Laos and Thailand on the Pak Beng hydropower project. The research focused on understating how affected local communities responded to the hydropower project and how these responses were linked with the way consultation was conducted by the company, and the role of external parties in this process. It also examined how these responses affected, and were affected by, intra – and inter-village relations and networks (Suhardiman, Manorom, and Rigg 2022).

Box 5. **The role of civil society organisations in transboundary water governance: Bridging scalar disconnects through inter-spatial co-learning.**

Civil society organisations under the Chiang Khong Conservation Group (CKCG) and Thai Mekong People’s Networks from Eight Provinces (TMNEP) in collaboration with local communities in specific localities play an important role in bridging the current scalar disconnects in transboundary water governance and initiating cross communities co-learning processes (Suhardiman, Manorom, and Rigg 2022). In the Mekong region, this is most apparent from these networks’ role in connecting local communities living along the river who are and will be affected by hydropower dams development. They did this through close collaboration with local communities regarding impact assessment, co-investigating with different groups within the communities how the dam construction will affect their livelihood options. Such collaboration resulted in the formation of inter-villages networks to share information about the dam planning. These networks function as grassroots decision-making structure to mobilise social movements, and serve as translocal knowledge assemblages between civil society organisations and the local communities, and among local communities themselves. Civil society organisations visited local communities in various localities surrounding hydropower dam construction sites in Laos but also including those resided in Thailand to promote grassroots cross-learning. Here, they are in fact activating the nodes of riverine grassroots movements across spatial scales and serve as knowledge broker. They rely on local communities’ views of the riverine ecosystems, how these have changed over time in relation to hydropower dam building, and support their ability to create strategic alliances within and across national borders. While processes of co-learning and sharing of information and experiences emerge from this and resulted in local communities’ strengthened mobilisation skills to resist national government’s development plans, such resistance is often scattered, short-lived and limited to particular localities. This is mostly because the company could use its power to break down these grassroots riverine alliances by using political force or favouring some groups within local communities at the expense of others, and thus dividing local communities’ common interests at its core.

## Final remarks

Land, and water-based multispecies cultures that integrate human and nonhuman societies and live generatively in riverine environments, including amphibious societies, are disappearing due to current extractive development patterns. Therefore, in many places, protecting river socioecological systems is at the centre of the struggle towards planetary justice. This endeavour requires radically different paradigms of knowledge, methodologies, and ways of understanding our relationship with others, both humans and non-humans, breaking with the anthropocentric and androcentric worldview that is reified through the dichotomous logic of modernist science and techno-capitalist river interventions. Now more than ever, academia is called upon to co-create and put knowledge in service of social, political, and environmental action.

In this paper we have elaborated the notion of river co-learning arena (RCA) as a possible way to do so. For this, we have first sketched the complex context in which RCAs operate, and then argued about the necessity and potential of solidary partnerships between social movements and activist scholars through action research practices such as RCAs. Then, we have outlined five “fields of action” of RCAs, which respond and counteract some of the challenges associated with the multifaceted landscape of social mobilisations to address river related problems, as well as with multistakeholder encounters as such. We have illustrated each of these aspects, their challenges and actions needed to address them within the RCA through an illustrative case box. Table 1 provides a summary of these insights and illustrations. It goes without saying that each of the challenges and corresponding implications and actions will strongly depend on, and vary according to, the social, political and environmental context of each RCA, the actors involved, and available resources.

**Table 1.** Summary of key aspects included in RCAs.

RCA challenge	RCA action	Box
Identifying and contesting dominant truth regimes and subjugated knowledges (and how they are disqualified)	Creating counter-truths and valuing alternative knowledge	1: Thinking and knowing natures and rivers “otherwise”
Moving beyond a traditional empirical approach to knowledge creation and developing new river understandings through relational co-learning	Deploying highly experiential and immersive methods that integrate sensing, feeling and thinking	2: Transgressive co-learning experiences through countermapping
Going beyond merely resolving opposing perspectives through fabricated participatory consensus and harnessing the transformative potential of political contention within RCAs	Recognising ontological pluralism and engaging reflexive dialogue	3: The tides of confrontation and collaboration in the case of Santurban Páramo in Colombia
Moving beyond anticipatory reflexivity and acknowledging the influence of positionality in RCA interactions and emerging subjectivities over the RCA processes	Applying an active and ongoing reflexivity stance through various processes of self and group reflection	4: Shifting perspectives on rivers as ecosociety through reflexivity in action research in Colombia
Translocal bridging of dispersed RCAs that share converging issues, concerns, interests, and practices and enable translation processes to foster bridging while respecting differences	Setting up virtual or material ways to support RCA network formation	5: The role of civil society organisations in bridging scalar disconnects through translocal co-learning

The presented examples suggest that much can be learned from engaging with and in those RCAs that strive for more holistic and renewed ethical relationships with water and commons. Through problematising and subsequently addressing asymmetric relations, ontological divides and historical legacies, they can enhance transformative processes of riverine co-production and more socio-environmentally just forms of river governance and stewardship.

As we outlined in the introduction, RCAs are not a format or blueprint that exists “out there”, that is set in stone, ready to be followed and replicated. Rather, the notion of RCA is a hybrid between on the one hand, diverse forms of collaboration, co-*creation* and co-learning that are taking place around the world in different shapes and styles as we speak; and, on the other hand, conceptual–theoretical considerations arising from these very hands-on experiences as well as from related academic-activist discussions. The resulting notion and reflections are thus an open invitation to bravely engage in activist research, encourage ontological and epistemological pluralism, and set-up new forms of trandisciplinary, reflexive knowledge co-creation.

Of course, this is far from an easy undertaking, as we have emphasised time and again. Even though we hope and believe that this contribution may provide some valuable orientation for reflection, challenges remain. For example, there is still the important question of the temporal permanence of RCAs to realise their desired outcomes. In fact, we consider that RCAs should best be seen as transient configurations that last as long as the energy balance of the learning configuration remains positive, i.e. people get more energy and results out of their participation than they put into it. This criterion refers to the phenomenon whereby individuals begin to withdraw from a particular endeavour due to waning interest, shifting priorities, lack of discernible progress or, to the contrary, accomplished objectives and results. A successful RCA recognises the appropriate point at which to end its activities. This seems counterintuitive and differs from conventional expectations of innovation processes, where people typically strive to maintain momentum and explore ways to scale up. However, rather than to keep an effort going, new creative routes and actions may be developed. As important is our learning that energy spent and capacities built in particular RCAs are not “wasted” when its structures wane: what we found is that grassroots leaders, and others empowered, quickly build and decisively engage in newly emerging networks and RCAs, to further deploy their enhanced capacities, skills and knowledge. Redirecting the energy expended in maintaining existing initiatives to initiate new RCAs from alternative perspectives will often also serve to engage new actors.

Thus, RCAs require much courage: to end but also to set up or engage with in the first place, to leave comfortable spaces, languages and perspectives behind and instead engage with contentions, uneasiness and unknown waters. At the same time, we believe this courage will not remain unrewarded: numerous grassroots initiatives have shown the potential of uniting in struggle, of daring to experiment with new forms of collaboration, learning, and boundary-crossing. We hope that in the coming years, more and more examples of RCA-like platforms emerge that jointly conduct research, co-produce knowledge and design action-oriented strategies that contribute to river regeneration and socio-ecological justice.
